# Application of Neural Network in Predicting H_2_S from an Acid Gas Removal Unit (AGRU) with Different Compositions of Solvents

**DOI:** 10.3390/s23021020

**Published:** 2023-01-16

**Authors:** Mohd Hakimi, Madiah Binti Omar, Rosdiazli Ibrahim

**Affiliations:** 1 Department of Chemical Engineering, Universiti Teknologi PETRONAS, Seri Iskandar 32610, Malaysia; 2 Department of Electrical and Electronics Engineering, Universiti Teknologi PETRONAS, Seri Iskandar 32610, Malaysia

**Keywords:** acid gas, concentration of H_2_S, automated prediction, artificial neural network, multiple linear regression, Levenberg–Marquardt, scale conjugate gradient

## Abstract

The gas sweetening process removes hydrogen sulfide (H_2_S) in an acid gas removal unit (AGRU) to meet the gas sales’ specification, known as sweet gas. Monitoring the concentration of H_2_S in sweet gas is crucial to avoid operational and environmental issues. This study shows the capability of artificial neural networks (ANN) to predict the concentration of H_2_S in sweet gas. The concentration of N-methyldiethanolamine (MDEA) and Piperazine (PZ), temperature and pressure as inputs, and the concentration of H_2_S in sweet gas as outputs have been used to create the ANN network. Two distinct backpropagation techniques with various transfer functions and numbers of neurons were used to train the ANN models. Multiple linear regression (MLR) was used to compare the outcomes of the ANN models. The models’ performance was assessed using the mean absolute error (MAE), root mean square error (RMSE), and coefficient of determination (R^2^). The findings demonstrate that ANN trained by the Levenberg–Marquardt technique, equipped with a logistic sigmoid (logsig) transfer function with three neurons achieved the highest R^2^ (0.966) and the lowest MAE (0.066) and RMSE (0.122) values. The findings suggested that ANN can be a reliable and accurate prediction method in predicting the concentration of H_2_S in sweet gas.

## 1. Introduction

In recent decades, there has been a sharp increase in the demand for natural gas. Despite its significant contribution to the current global economy and progress, natural gas typically contains a number of pollutants that must be removed in order for it to meet the requirements for gas pipelines, such as acid gases (hydrogen sulfide (H_2_S) and carbon dioxide (CO_2_)) [1]. Due to human health and environmental concerns, numerous restrictions and regulations have been imposed on the gas sales’ specifications. The permitted concentration of H_2_S is between 4 and 20 part per million (ppm) and not more than 3% of CO_2_ [2]. Therefore, the acid gas must undergo a gas treatment process, namely gas sweetening. In the gas sweetening process, acid gasses, such as H_2_S and CO_2,_ are removed to produce a sweet gas [3].

The gas sweetening processes can be divided into several categories, which include physical, chemical and hybrid solvents, adsorption processes and physical separation. Alkanolamine solvents for absorption have been the most extensively utilized commercial technology in a variety of industries in recent years [2]. Utilizing the solvents’ capacity and reducing the process’s operating costs are two common strategies put forth by researchers. Studies have shown that mixed tertiary amines, such as N-methyldiethanolamine (MDEA) and tri-ethanolamine (TEA), showed a positive impact as they reduced the process costs by up to 3% when 40% MDEA and 5% TEA were used [4]. According to Ammar and Samah (2020), a formulated solvent known as mixed amines, which contains MDEA as the primary solvent, is typically blended or added with one or more reactive amines such as piperazine, TEA, sulfolane, or diethanolamine (DEA) [2].

The purpose of mixing different amines is to combine each of their favourable characteristics to maximize the removal capacity of the acid gas. In contrast to primary and secondary amines, the tertiary amine MDEA has a lower vapour pressure, greater resilience to deterioration, and fewer corrosion issues [5]. Other than these advantages, the disadvantage is that it does not directly react with CO_2_, slowing down the absorption rate of CO_2_ in aqueous MDEA [6]. To increase the rate of absorption, activators, including piperazine (PZ), monoethanolamine (MEA), and sulfolane, are used with MDEA [7]. Using PZ as an activator has shown positive results in the past few years. CO_2_ is absorbed more quickly by MDEA when PZ is added than by MEA or DEA [8]. One study that established a detailed model to examine the solubility of CO_2_ into activated MDEA by PZ demonstrated that theoretically one mole of PZ can absorb two moles of CO_2_ [9].

It is necessary to establish the lean amine mixture’s ideal concentration, which matches the H_2_S and CO_2_ levels in sweet gas. A typical alkanolamine-based solvent ranges from 30 to 45 wt% MDEA and 5–20 wt% piperazine, with the rest made up of water [10]. Still, there is no guarantee of optimal removal efficiency for such a prescribed solvent. Given the increasing range of feed gas conditions and varying process operations, such as those encountered in current gas field practice, there is a need to consider a systematic approach for optimizing such solvents [11]. Artificial neural networks (ANNs) have been widely used in this context to solve engineering applications, particularly for the prediction of highly non-linear systems [12]. The growing body of research on ANN applications has demonstrated its advantages over traditional regression. This might be a result of how closely these networks resemble the human brain [13]. There are three layers that make up the ANN architecture: input, hidden, and output. Neurons make up for each of the layers and are linked by synapses with weighted coefficients [12]. 

When compared to other ANN types, the multilayer perceptron (MLP) with a backpropagation learning approach is employed the most frequently for problem-solving [14]. Several studies have shown the use of ANN in natural gas treatment. A study on the application of ANN in predicting the output parameter of gas sweetening regeneration column revealed that the developed ANN model produced a good consistency with the experimental data, which concluded that the model developed can be used for predicting the output parameters accurately [15]. Another study regarding predicting the solubility of H_2_S for both single and blended adsorbents has shown that the ANN model with 17 hidden neurons produced the highest R^2^ (0.9817) and the lowest mean square error MSE (0.0014) [12]. Furthermore, the experimental data and the predicted solubility of H_2_S is well aligned.

In order to find the optimum prediction model for predicting the H_2_S content in sweet gas, an ANN model based on MLP with various transfer functions and number of neurons was created for this study. The outcomes of ANN have also been evaluated with those of multiple linear regression (MLR). Section 2 of this paper describes the natural gas sweetening plant, flowsheet development and validation and proposed system models for predictions. Section 3 in this paper provides the results and discussion and is followed by the conclusion of this study in Section 4.

## 2. Materials and Methods

**Figure 1:**
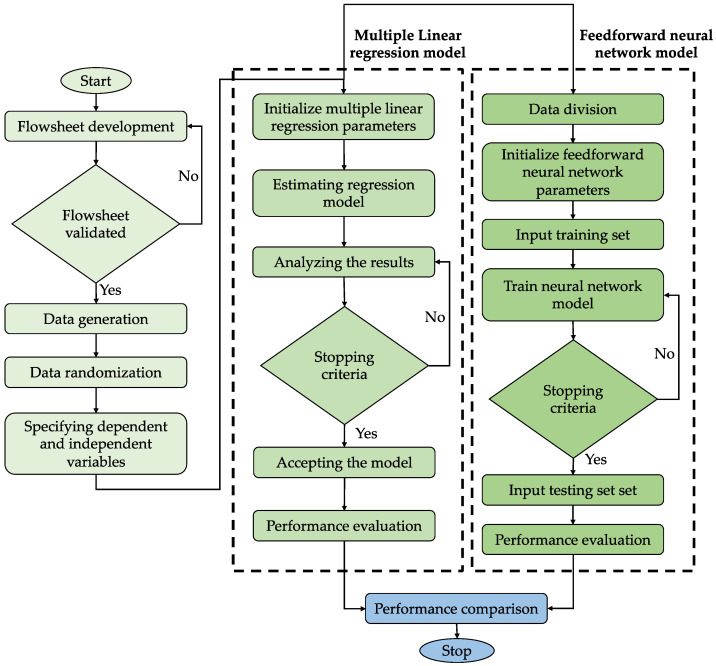
Overall flow design of this study.

The methods used to create the models that predict the H_2_S content of sweet gas are discussed in this section. MLR and ANN were used to train the data from a validated simulation. In contrast to linear regression, which was compared in this study due to its ease and widespread use in many applications and research, ANN was chosen due to its greater recognition and superior performance in predicting non-linear correlations. Figure 1 depicts the overall flow of the study’s work plan.

### 2.1. Process Description of Natural Gas Sweetening Plant

As mentioned, there are many methods used to treat acid gas. However, the amine process is currently the most widely used method in acid gas removal [15]. The reversible reaction for the gas sweetening process is as follows [16]:

2RNH_2_ + H_2_S ⇋ (RNH_3_)S (1)

2RNH_2_ +CO_2_ ⇋ (RNH2)2H_2_CO_3_ (2)

where R is mono-, di- or tri-ethanol, while N, H, S, C and O represent nitrogen, hydrogen, sulphur, carbon and oxygen, respectively. The forward reaction of this process is exothermic, and its reversible reaction is endothermic. The endothermic reaction of this process can be promoted by heating the amine solution.

**Figure 2:**
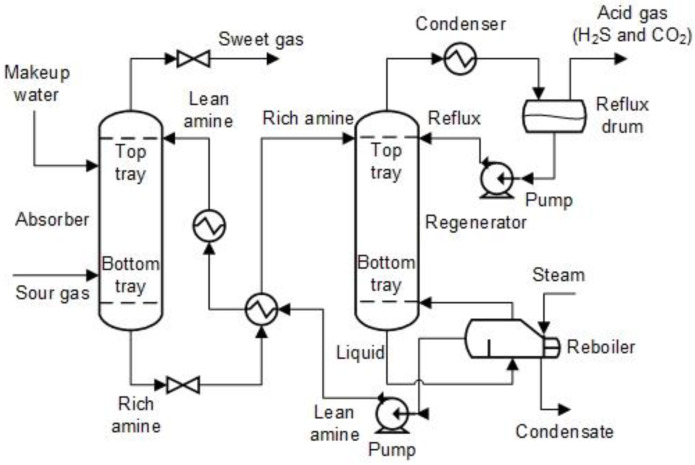
Typical process flow diagram of an amine-treating unit.

A typical process flow diagram for an amine-treating unit is shown in Figure 2. A simplified version of the process description is provided below to facilitate further discussion; the complete process description is also available from [17]. The sour feed gas is typically supplied into an input separator first to be cleansed and filtered of any free liquids and solids before being fed into the amine unit. The aqueous amine solution enters at the top of the amine absorber unit, and the sour gas is then fed into the bottom, flowing counter-currently while in contact with it. H_2_S in the gas phase is transported to the liquid phase during the absorber contact period in accordance with the reaction of Equation (1). The amine solution that absorbed the acid gas and emerged from the bottom of the absorber is the rich amine. Sweet gas is defined as sour gas that has been treated in an absorber where the H_2_S concentration has dropped to below the standard limit of 4 ppm and not more than 3 percent mole of CO_2_ leaves at the absorber’s top.

The rich amine flows into a flash separator at low pressure, removing the hydrocarbons through weight differences and separating gas components. Before entering the regenerator, the rich amine is pre-heated through a lean/rich heat exchanger, reducing the reboiler’s heat load. This is a preliminary step in removing H_2_S. When the rich amine is in touch with the high-temperature amine vapour phase created in the reboiler, regeneration takes place in the regenerator. As previously mentioned, a high temperature encourages the reversal of Equation (1). Lean amine, which has a high temperature and a low percentage of acid gas, is then cycled back to the absorber. In order to recover the vaporised amine, the rich amine’s stripped acid gas is cooled in a condenser before being refluxed back into the regenerator. Before they may be released into the atmosphere without harm, the acid gas that leave from the reflux’s top needs to undergo additional processing.

### 2.2. Sour Feed Gas Specification

Before moving forward with the data generation of MDEA and PZ, a validated flowsheet is required. Some of the conditions of the variables are adopted from the previous literature. The flow rate of the sour gas, its pressure and temperature are 7154.8 kmol/h, 54.03 bar and 52 °C. MDEA is commonly used between 30 to 50 wt%. As for this simulation, 48% of MDEA is used to simulate and compare with industrial data obtained from the previous literature [18]. The MDEA solution enters at the top of the absorption tower at a pressure and temperature of 54.32 bar and 55 °C, respectively. Table 1 shows the sour feed gas composition used for the simulation.

### 2.3. Simulation Validation

**Figure 3:**
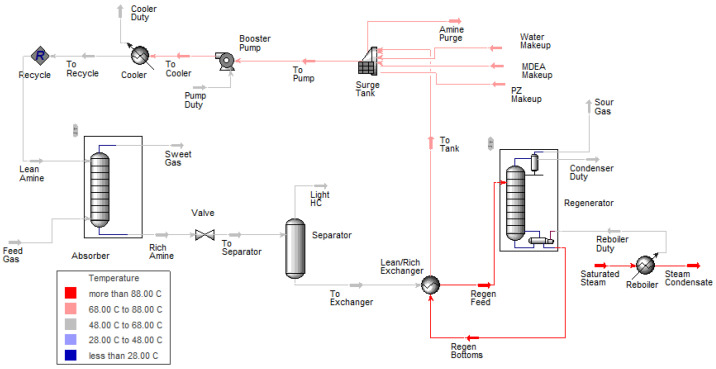
Process flowsheet for acid gas treatment.

A case study was developed to validate the simulation results’ accuracy and reliability, which will then be used for data generation. The simulation was developed in Aspen HYSYS 12.1 with an acid gas fluid package, which is recommended for natural gas treatment [2]. Figure 3 shows the developed flowsheet for acid gas treatment. Table 2 shows the simulation and the industrial data’s flow rate of H_2_S and CO_2_ in sweet gas. The error comparison between the industrial and simulation data is calculated as follows:

(3) $$\mathrm{Error}\;\left(\%\right)=\frac{\mathrm{Industrial}-\mathrm{Simulation}}{\mathrm{Industrial}}\times 100$$

The actual comparison of the flow rate of H_2_S, CO_2_ and the total flow of sweet gas between the industrial, the previous literature and simulation data show a reasonably good agreement. Thus, the flowsheet can be used for data generation.

### 2.4. Data Generation

From the converged simulation and validated flowsheet, numerous data can be generated by simulating different values of a single variable using the Aspen Simulation Workbook. The choice of parameters and the range of manipulation for each parameter are decided based on the previous literature. These datasets are crucial as data-driven models depend heavily on a large dataset. The larger the dataset, the better the model. The data produced are further utilized to study the effects of manipulating several operational parameters of the system. Above all, data generation is conducted in three steps: simulation of acid gas treatment using a base case scenario, identification of input and output variables, which are the independent and dependent variables and rigorous simulation using multiple case scenarios involving the manipulation of input variables and obtaining the corresponding output variables.

In machine learning, identifying independent and dependent variables is vital to make predictions using data-driven models. Independent variables are input variables that do not depend on other variables but might cause changes in other variables. In contrast, dependent variables are output variables solely influenced by the changes implied in the independent variables. The determination of independent and dependent variables is purely supported by the literature where standard input and output variables are identified. Their corresponding lower and upper boundaries for generating outputs are based on various manipulated input variables. As for the MDEA and PZ, their lower and upper limits were obtained from a previous study [2]. As for the temperature and absorber pressure, the output was obtained through a sensitivity analysis. Sensitivity analysis analyses how a certain dependent variable is affected by a changed value of an independent variable under a specific set of assumptions. The range of the temperature and absorber for this work is set to be ±30% of the base value. Table 3 shows the independent and dependent variables corresponding to lower and upper limits. Table 4 shows the framework for the data generation with respect to their input and their combination of inputs.

### 2.5. Data Collection and Pre-Processing

A total of 15 simulation runs have been conducted with respect to their input and their combination. This generates a total of 3015 data points from the data generation section. The parameters reported in this study are the essential variables that affect the rate of absorption of H_2_S. The concentration of MDEA and PZ, the temperature of amine solvent and the pressure of the absorber are the input variables, and the concentration of H_2_S in sweet gas is the targeted output. A total of five combinations of outputs were considered to determine the concentration of H_2_S in sweet gas, shown in Table 5. The predictive models were developed by using MATLAB version R2022a [14]. First, the code for the neural network was developed by utilizing the neural net fitting toolbox in MATLAB. Then, different transfer functions were tested by altering the codes. Next, the function “fitlm” was used for linear regression to develop the regression model. Finally, correlation matrices were plotted through a scatter plot before training to analyse the relationship between the variables.

### 2.6. Model Development

#### 2.6.1. Feedforward Neural Network-Based Model

**Figure 4:**
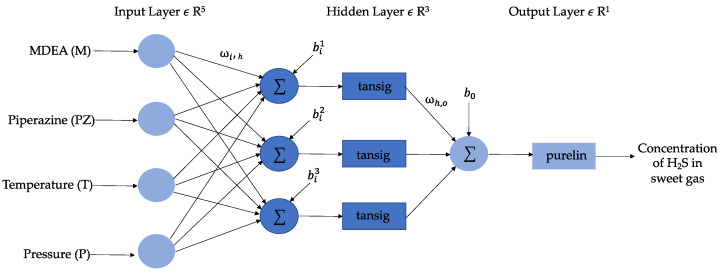
Feedforward neural network developed in this study.

A neural network is an algorithm that draws inspiration from the human brain and uses examples, patterns, and scenarios to learn and then applies them to solve problems [14]. The layers of neurons that make up a neural network serve as the network’s central processing units. In contrast to a model based on regression, ANN can handle noisy and non-linear data. Due to its simplicity of construction and ability to serve as a universal approximator, this study opts for a multilayer feedforward network or multilayer perceptron (MLP) with a single hidden layer [19]. A conceptual diagram of the created MLP used in this study is shown in Figure 4. The network receives inputs in its input layer, which has four input neurons, and predicts results in its output layer. Between the input and output, it has hidden layers where the majority of the network’s processing is needed and conducted. As an illustration, the hidden and output layer activation functions were performed using “tansig” and “purelin.” The MLP must be taught to recognise the ideal weights of connections between the neurons in order to obtain a minimal difference between the measured and predicted values of the dependent variable [20]. The most common method of training MLP is through a two-step approach of the backpropagation (BP) method. The first step is propagating the input signal to estimate the outputs [19]. In the second step, the weight vectors and biases between the layers are adjusted, the mean square error (MSE) between the observed and predicted results is reduced, and the model’s generalization is increased to make it more dependable [14]. 

#### 2.6.2. Multiple Linear Regression (MLR)

Regression analysis is a crucial statistical tool that determines the correlation between the dependent (response) and independent (predictors) variables. Linear regression is one of the simplest yet most widely used models in determining the strength between the predictors and the response. Regression can be divided into simple linear regression (SLR) and multiple linear regression (MLR). In SLR, the response (outcome) is predicted based on a single independent variable, which is depicted in (1). MLR is another name for a statistical tool that is immensely useful in figuring out the optimal correlation between a dependent variable and a number of independent variables. Since there are multiple inputs in this investigation, a linear equation was fitted to the data as a primary building block for correlation. The MLR models were created using the four model specification parameters, namely linear, interaction, pure quadratic, and quadratic, which are given in (4)–(8) [21].

(4) $${y=a}_{0}{+a}_{1}x+\epsilon$$

(5) $${y=a}_{0}+\overset{m}{\underset{i=1}{\sum }}a_{i}x_{i}+\epsilon \;$$

(6) $${y=a}_{0}+\sum_{i=1}^{m}a_{i}x_{i}+\sum_{i,j=1;i<j}^{m}a_{\mathrm{ij}}x_{i}x_{j}$$

(7) $${y=a}_{0}+\sum_{i=1;p<3}^{m}a_{i}x_{i}^{p}$$

(8) $${y=a}_{0}+\overset{m,p<3}{\underset{i=1;p<3}{\sum }}a_{i}x_{i}^{p}+\overset{m}{\underset{i,j=1;i<j;p,q<3}{\sum }}a_{\mathrm{ij}}x_{i}^{p}x_{j}^{q}+\overset{m}{\underset{i,j=1;i\neq j;p,q<3;p<q}{\sum }}a_{\mathrm{ij}}x_{i}^{p}x_{j}^{q}$$

where y is the predicted output, x, x_i_ and x_ij_ are the independent input variables, a_0_ is a constant term, a_i_ and a_ij_ are the regression coefficients, and ϵ is the residual error.

### 2.7. Model Training and Testing

To discover the model that best predicts values that are as close to the actual concentration of H_2_S in sweet gas as possible, model training was carried out using a variety of network configurations and learning parameters. Throughout the study, the data division for ANN and MLR was kept constant to compare their performances on equal grounds. Typically, 80% of the data is utilized for training and validation, and 20% is used for testing or verification [15]. Testing (verification) is used to evaluate the accuracy of the previously trained network by providing a dataset that has never been seen before by the network. Two types of BP algorithm, Levenberg–Marquardt (LM) and scale conjugate gradient (SCG) were involved in training the ANN models. Three distinct transfer functions, tangent sigmoid (tansig), logistic sigmoid (logsig), and radial basis function (radbas), were utilized to evaluate and optimise the ANN transfer function in the hidden layer. These functions have one thing in common: they all need to calculate e^x^ [22]. The equation of logsig, tansig and radbas are given in (9)–(11), where x is the independent variable and j = 1, 2, …, 5. Meanwhile, the activation function for the output layer is set to be a linear transfer function, which is purelin for all models. This function returns the value passed to the neuron to calculate its output.

(9) $$f\left(x\right)=\frac{1}{{1-e}^{-x^{\prime }}}$$

(10) $$f\left(x\right)=\frac{e^{x}{-e}^{x}}{e^{x}{-e}^{-x^{\prime }}}$$

(11) $$f\left(x\right)=\exp(-\frac{1}{{2\sigma }_{j}^{2}}{\left|{x-x}_{j}\right|}^{2})$$

**Figure 5:**
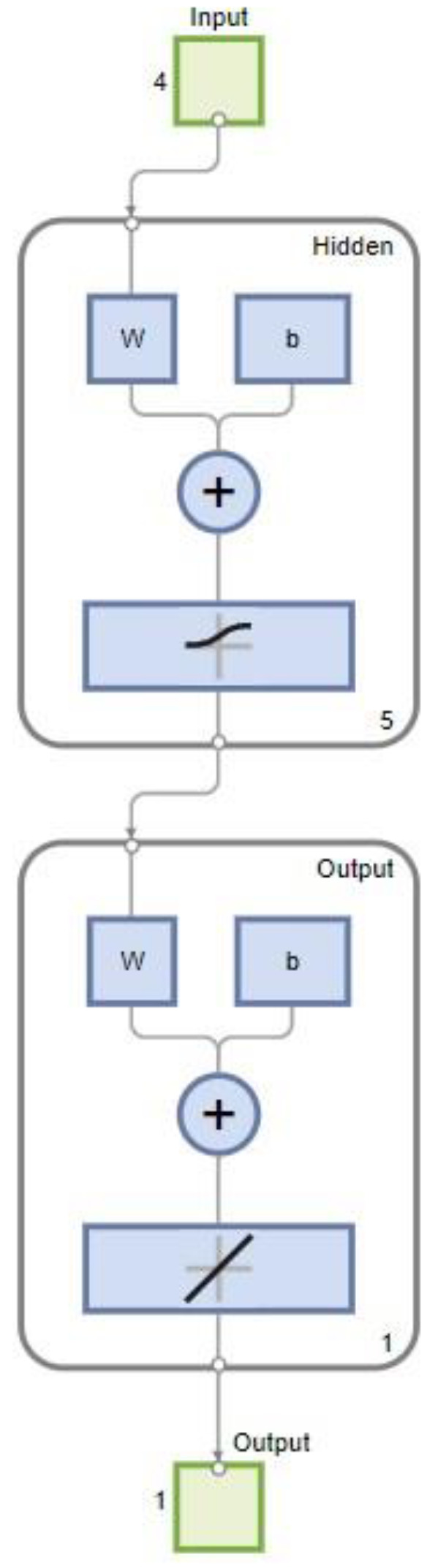
Feedforward neural network model.

The feedforward neural network with a single hidden layer used in this paper is shown in Figure 5. The first layer of the network model in the model has five nodes to represent the inputs (M, PZ, T, and P), while the last layer has one node to represent the output (concentration of H_2_S in sweet gas). The five-node hidden layer is represented by the middle layers. For instance, logsig and purelin were utilized as the activation functions for the hidden and output layers, as shown in Figure 5. Usually, purelin is used as the transfer function for the output layer. The combination of non-linear and linear transfer functions has achieved efficient training from a previous study [23]. The number of neurons was another criterion that was changed in this study. Input and output layer neurons should be equal in number to the number of independent and dependent variables [14]. Nevertheless, for determining the minimum and maximum number of neurons in the hidden layer, there are no established standards [24]. The number of neurons selected is crucial because too few neurons could make network learning more complex and too many neurons would need excessive training time. On the other hand, for testing the MLR models, the equations obtained through training are used by substituting the testing dataset. 

### 2.8. Model Performance and Evaluation

The performance of the developed ANN model and the MLR models in predicting the concentration of H_2_S in sweet gas were evaluated through mean absolute error (*MAE*), root mean square error (*RMSE*) and coefficient of determination (*R*^2^). The differences between the actual and predicted outputs are commonly represented by the metrics *MAE*, *RMSE*, and *R*^2^ (in this case, the actual and predicted concentration of H_2_S in sweet gas). An ideal model should have the value of *MAE* and *RMSE* approaching zero, while *R*^2^ converges to unity [25]. Calculations of the three predictive indicators (*MAE*, *RMSE* and *R*^2^) are defined as follows [14]:

(12) $$MAE=\frac{1}{n_{s}}\overset{n_{s}}{\underset{k=1}{\sum }}\left(Y_{A,k}-Y_{P,k}\right)$$

(13) $$RMSE=\sqrt{\frac{1}{n_{s}}\overset{n_{s}}{\underset{k=1}{\sum }}{\left(Y_{A,k}-Y_{P,k}\right)}^{2}}$$

(14) $$R^{2}=\frac{\sum_{k=1}^{n_{s}}{\left(Y_{A,k}-Y_{P,k}\right)}^{2}}{\sum_{k=1}^{n_{s}}{\left(Y_{A,k}-Y_{AVG}\right)}^{2}}$$

where *n_s_* is the number of the data points, $Y_{A}$ is the actual H_2_S concentration and $Y_{P}$ is the predicted H_2_S concentration.

## 3. Results and Discussion

**Figure 6:**
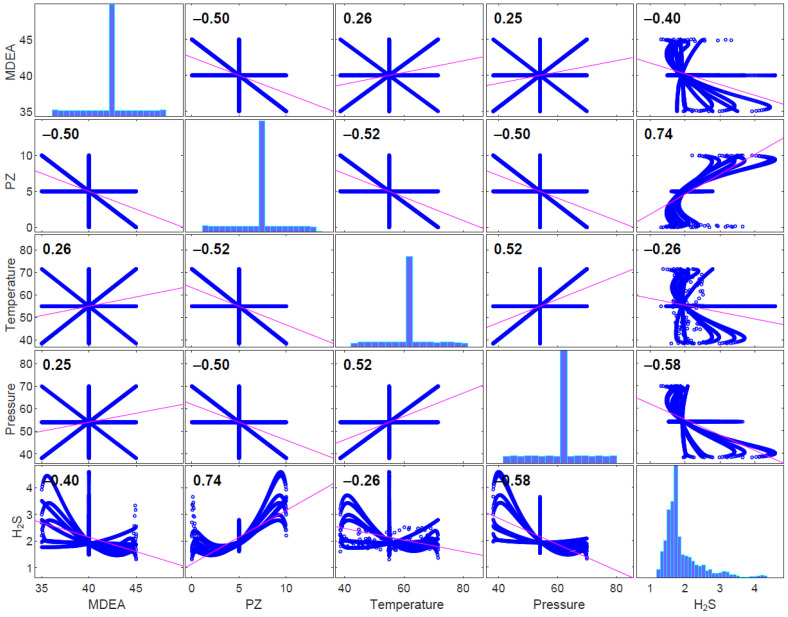
Scatter plots of concentration of H_2_S in sweet gas versus four parameters: MDEA (M), Piperazine (PZ), temperature (T) and pressure (P).

The correlation matrix shown in Figure 6 was used to examine the correlations between all factors. The response, or the concentration of H_2_S in sweet gas, and the predictors, namely MDEA, PZ, temperature, and pressure, could be seen to be linear. According to [26], the range of the correlation coefficient is 0.70–0.89, signifying a strong correlation; 0.40–0.69, a moderate correlation; and 0.10–0.39, a poor correlation. From Figure 6, it can be observed that PZ has a strong correlation (0.74) with the concentration of H_2_S in sweet gas, followed by pressure and MDEA, which is considered a moderate correlation and temperature (−0.26), which indicates a weak correlation. Additionally, no multicollinearity is observed between the predictors. Multicollinearity is a condition that includes a significant degree of correlation between two or more independent variables (predictors), which could raise the standard error of the coefficient. If this happens, some factors may not be statistically significant even if they ought to be [27]. ANN and MLR models were used to further study the relationship between the H_2_S content in the sweet gas and the input parameters. MAE, RMSE and R^2^ were used to evaluate their performances for this study.

### 3.1. Performances of ANN Models

The performance of the developed ANN models can be evaluated through Table 6. From Table 6, it can be observed that the network structure with an LM algorithm equipped with logsig transfer function and five neurons shows the best prediction accuracy compared to the others. Model 3’s combination of three predictors (PZ concentration, temperature, and pressure) proved to be the most effective in this structure. The MAE and RMSE values for the dataset were 0.066 and 0.112, respectively, while the R^2^ determination coefficient for the dataset was 0.966, which is the highest value. The fraction of model predictions that matches the data increases as R^2^ increases. Additionally, it shows that the interpretation of PZ, temperature, and pressure can account for at least 96% of the variation in H_2_S concentration in sweet gas, indicating the usefulness of utilizing ANN in this study. It is important to note that the models’ accuracy increases as the number of neurons rise from one to five. This can be supported by the decreasing values of MAE and RMSE as the number of neurons increases.

Between ANN-LM and ANN-SCG, LM showed better consistency in terms of prediction accuracy. The prediction accuracy increases as the number of neurons increases. This can be due to the increasing number of synapses used for learning and data fitting [12]. The accuracy of the SCG method does not appear to be correlated with the number of hidden neurons, as demonstrated by the inconsistent prediction accuracy values that SCG showed as the number of neurons increased. The conjugate directions and scaling of step size are used to create and derive the SCG algorithm [28], resulting in the algorithm being ideal for a large-scale, extremely non-linear prediction of thousands of data points [12]. The LM algorithm, on the other hand, addresses problems by iteratively altering a weighted computation until an acceptable answer is found. It is a non-linear optimization and minimisation of prediction error. Therefore, this algorithm is suggested to be suitable for complex–medium scale problems [29]. In the present study, with 3015 data points, a relatively superior prediction performance of ANN-LM is observed as compared to ANN-SCG.

The impacts of various transfer functions were also thoroughly studied. Although theoretically, neural networks might learn any mapping, they could require flexible “brain modules” or transfer functions that are suitable for problem-solving [30]. In comparison to tansig and radbas, it achieves a higher accuracy by utilizing the logsig transfer function with five neurons. The radbas transfer function, followed by logsig and tansig, yields superior results for the SCG algorithm. Each transfer function has its own advantages. As the logsig transfer function is differentiable, it is often employed in multilayer networks trained with the backpropagation technique [31]. Tansig decreases the chance of neuron saturation by providing stronger gradients than logsig. Additionally, it avoids the “biassing” of gradients and speeds up the training of backpropagation algorithms for networks with an extensive amount of connectivity, which can be seen in [32]. Radial basis functions (RBFs), on the other hand, have a tendency to learn more quickly because each RBF unit only covers a small portion of the input space. It works well in higher layers that are low-dimensional and in lower layers that are high-dimensional sigmoid [33].

The findings in this study are similar with the findings in [12], where ANN is implemented to predict the solubility of H_2_S since obtaining the experimental solubility of H_2_S is time consuming and costly. Temperature, pressure, adsorbents and their weight fractions are some factors that influence the solubility of H_2_S. Different numbers of neurons were used to compare three methods, including LM and SCG. Mean square error (MSE) and determination of coefficients (R^2^) was used to evaluate the performance of the models. With 17 neurons, LM-ANN achieved the most astounding prediction performance of H_2_S solubility throughout the investigation, with MSE of 0.0014 and R^2^ of 0.9817 as compared to SCG-ANN (0.0791,0.8626).

Furthermore, the effect of input parameters on the concentration of H_2_S in sweet gas was also investigated in this work. It can be observed that as the inputs change, the performance of the ANN models with various transfer functions changes. The results obtained using Dataset 4 showed lower accuracy, with a lower coefficient of determination (R^2^) and higher error values for both algorithms. The input parameter, Piperazine (PZ), is significant (0.74) towards the concentration of H_2_S in sweet gas, which is supported by the scatter plots in Figure 6. This indicates that the importance of having PZ as an input parameter and the absence of PZ reduces the prediction accuracy. 

### 3.2. Performances of MLR Models

In this study, the effectiveness of ANN was compared to that of MLR. MLR was created based on four distinct model specifications: quadratic, interactions, pure quadratic, and linear. Table 7 displays the performance of each model. Higher range of R^2^ and lower range of MAE and RMSE resulted from regression through quadratic. The quadratic terms obtained the highest R^2^ value, 0.92, and the lowest, 0.53. Dataset 5, which contains all the input variables (MDEA, PZ, temperature and pressure), has shown the best performance in predicting the concentration of H_2_S in sweet gas, obtaining the lowest values of MAE and RMSE and the highest R^2^ value as compared to the other dataset. The MLR model with quadratic terms produced from Dataset 5 is shown in Equation (15).

(15) $$\mathrm{Concentration}\;\mathrm{of}\;H^{2}S\;\mathrm{in}\;\mathrm{sweet}\;\mathrm{gas}=3.2440+0.0174M-0.6617\mathrm{PZ}+0.0819T-0.0872P+0.0087\mathrm{MPZ}-0.0022\mathrm{MT}\;+0.0008042\mathrm{MP}+0.0040\mathrm{PZT}-0.0028\mathrm{PZP}-0.0009485\mathrm{TP}+0.00012292M^{2}+0.0431{\mathrm{PZ}}^{2}+0.00059214T^{2}+0.00081678P^{2}$$

In Table 7, from testing Dataset 4, which does not include PZ as the input parameter, it can be observed that all types of MLR gave low values of R^2^, ranging from 0.44 to 0.53. However, when PZ was incorporated as the input, all models’ accuracy improved, ranging from 0.59 to 0.92. This shows the significance of PZ as an input parameter towards the output. Furthermore, the accuracy of the models developed with different model specification increased in this order: linear (0.44–0.70), interaction (0.47–0.87), pure quadratic (0.51–0.91) and quadratic (0.53–0.92).

### 3.3. Comparison between ANN and MLR

**Figure 7:**
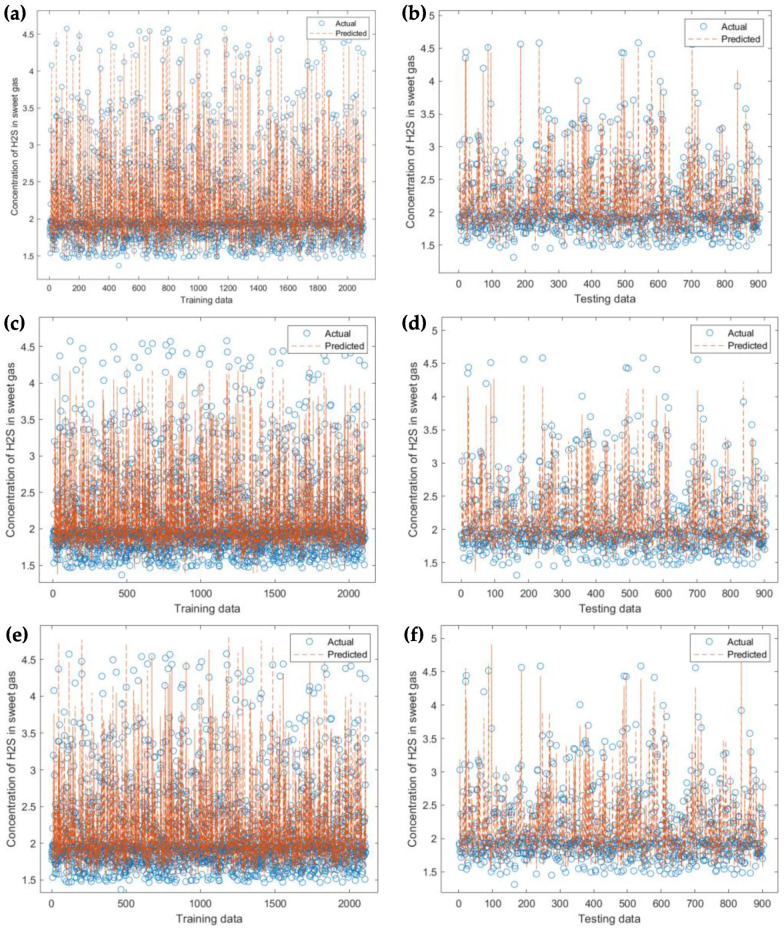
Comparison of actual and predicted concentration of H_2_S in sweet gas of (**a**) training and (**b**) testing of ANN-LM, (**c**) training and (**d**) testing of ANN-SCG, (**e**) training and (**f**) testing of MLR.

The results obtained from ANN and MLR models during training and testing have been compared to evaluate their performances. In terms of values, the performance of ANN-LM in predicting the concentration of H_2_S in sweet gas is better compared to the other models. The results from Figure 7a, which compares the actual and predicted values of the output during training, reveal that the model achieved a high R^2^ value of 0.96 and low MAE (0.06) and RMSE (0.11) values. During the training, the R^2^ value from ANN-LM was 0.96, indicating that the model follows almost perfectly the actual data. The developed model can achieve a higher prediction accuracy when test datasets are used, as seen in Figure 7b. ANN-SCG model also depicts a high accuracy by obtaining a high R^2^ value for training (0.92) and testing (0.94) (refer to Figure 7c,d). On the other hand, MLR with quadratic terms showed a lower value of R^2^ for training (0.91) and testing (0.91), indicating that the model is less reliable in predicting the concentration of H_2_S in sweet gas as compared to the other models. The accuracy of the models developed in this study increased in this order: MLR, ANN-SCG and ANN-LM. 

**Figure 8:**
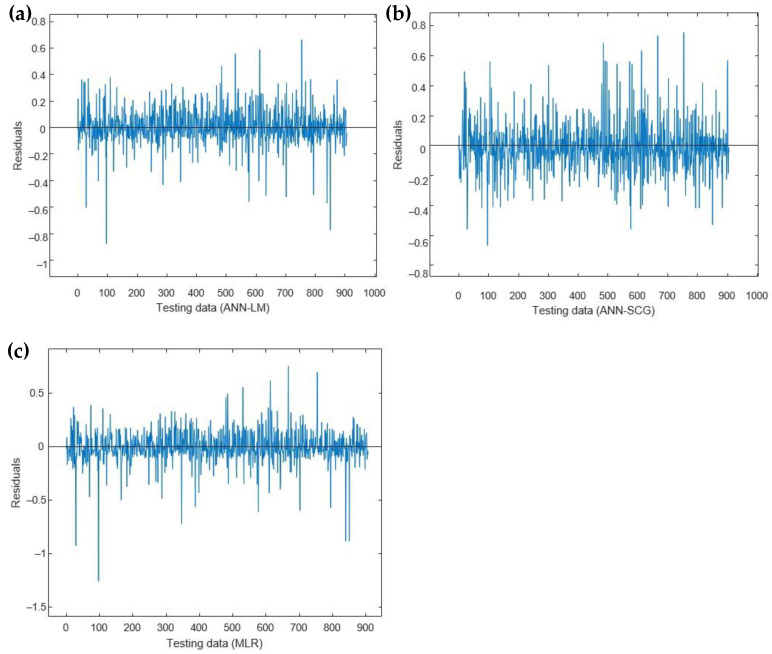
Residual comparison among (**a**) ANN-LM, (**b**) ANN-SCG, (**c**) MLR with the actual value of the concentration of H_2_S in sweet gas.

Figure 8 shows the residuals between each model’s actual and predicted data. The maximum residual for testing datasets for ANN-LM, ANN-SCG and MLR were determined to be around 0.87,0.76 and 1.25. These results reveal the capability of ANN in predicting the concentration of H_2_S in sweet gas, which provides better accuracy than MLR.

## 4. Conclusions

This work shows the capability of ANN in predicting the concentration of H_2_S in sweet gas, which is crucial for safety reasons and design requirements. To develop ANN and MLR models with high accuracy in predicting the concentration of H_2_S, the concentration of MDEA and PZ, temperature and pressure are used as the inputs, and H_2_S concentration in sweet gas is used as the output. Different learning methods, transfer functions and the number of neurons were investigated to obtain a suitable model. In this work, a model developed with three input parameters (Dataset 3), which include MDEA, PZ and pressure from Levenberg–Marquardt equipped with logsig transfer function with five neurons, showed the highest prediction accuracy, which obtained the highest R^2^ (0.966) value and the lowest MAE (0.066) and RMSE (0.122) as compared to the others. Throughout this study, the absence of PZ in the dataset reduced the prediction accuracy for both types of models, indicating the importance of PZ in predicting the concentration of H_2_S. Both models, ANN and MLR, could predict the overall desirability of concentration of H_2_S in sweet gas with relatively good adjustment and fit between 0.700–0.966. However, the ANN models showed better performance compared to MLR models. This study has shown the potential application of ANN as a modelling tool to predict the concentration of H_2_S in sweet gas in the oil and gas industry. This study contributes to the exploration of ANN as a prediction model for H_2_S in sweet gas. The utilization of ANN models as an alternative method throughout this work can be used as a context and guidance for the researchers and the industry. In future works, ANN could be utilized to determine the best composition of solvents and other sets of parameters for H_2_S absorption that reduce the time and cost for the intended solvent optimization study compared to conventional trial-and-error-based experimental methods.

## Figures and Tables

**Table 1:** Sour feed gas composition used for the simulation.

Composition	Mole Fraction
H_2_O	0.00234
i-Pentane	0.00520
n-Pentane	0.00560
i-Butane	0.01243
n-Butane	0.02150
CO_2_	0.03001
H_2_S	0.00024
Methane	0.72583
Ethane	0.11717
Propane	0.07377

**Table 2:** Comparison of H_2_S, CO_2_ and total flow of sweet gas between industrial, literature and our simulation data.

Properties	Industrial	Literature [18]	Error (%)	Simulation	Error (%)
H_2_S (kmol/h) *	0.00959	0.01001	4.37	0.0101	5.31
CO_2_ (kmol/h) *	170.87	169.41	0.85	171.09	0.13
Total flow (kmol/h) *	7031.80	7029.47	0.033	6974.00	0.82

* Units: Kilomol/hour (kmol/h).

**Table 3:** List of independent and dependent variables corresponding with their lower and upper limits for data generation.

Parameters		Lower	Base	Upper	Variables
MDEA (%) [2]		35.00	40.00	45.00	Independent
Pz (%) [2]		0.00	5.00	10.00	
Temperature (°C) [18]		38.50	55.00	71.50	
Absorber Pressure (bar) [18]	Feed	38.12	54.02	69.93	
	Lean amine	38.33	54.32	70.32	
	Top	37.30	52.85	68.41	
	Bottom	37.64	53.33	69.03	
Concentration of H_2_S (ppm) in sweet gas					Dependent

**Table 4:** Data generation framework with respect to their input and their combination.

Data Generation	Input			
	MDEA (%)	PZ (%)	Temperature (°C)	Pressure (bar)
1	35.00–45.00	Base	Base	Base
2	Base	0.00–10.00	Base	Base
3	Base	Base	38.50–71.50	Base
4	Base	Base	Base	37.30–70.32
5	35.00–45.00	0.00–10.00	Base	Base
6	Base	0.00–10.00	38.50–71.50	Base
7	Base	Base	38.50–71.50	37.30–70.32
8	35.00–45.00	Base	38.50–71.50	Base
9	35.00–45.00	Base	Base	37.30–70.32
10	Base	0.00–10.00	38.50–71.50	Base
11	Base	0.00–10.00	Base	37.30–70.32
12	35.00–45.00	0.00–10.00	38.50–71.50	Base
13	35.00–45.00	0.00–10.00	Base	38.50–71.50
14	Base	0.00–10.00	38.50–71.50	38.50–71.50
15	35.00–45.00	0.00–10.00	38.50–71.50	38.50–71.50

**Table 5:** Combination of input parameters in determining the concentration of H_2_S in sweet gas.

Dataset	Inputs	Outputs
1	Concentration of MDEA, PZ and temperature	Concentration of H_2_S (ppm) in sweet gas
2	Concentration of MDEA, PZ and pressure	
3	Concentration of PZ, temperature and pressure	
4	Concentration of MDEA, temperature and pressure	
5	Concentration of MDEA, PZ, temperature and pressure	

**Table 6:** Performance of ANN models with different transfer function and number of neurons during testing.

Number of Neurons			1			2			3			4				5	
Training Function	Transfer Function	Dataset	MAE	RMSE	R^2^	MAE	RMSE	R^2^	MAE	RMSE	R^2^	MAE	RMSE	R^2^	MAE	RMSE	R^2^
Levenberg–Marquardt	Logsig	1	0.187	0.257	0.797	0.175	0.250	0.809	0.165	0.241	0.822	0.158	0.229	0.839	0.164	0.231	0.836
		2	0.149	0.227	0.831	0.133	0.206	0.861	0.125	0.188	0.884	0.127	0.177	0.898	0.128	0.181	0.893
		3	0.117	0.179	0.914	0.105	0.153	0.937	0.085	0.137	0.949	0.078	0.138	0.949	0.066	0.112	0.966
		4	0.270	0.413	0.495	0.251	0.378	0.576	0.248	0.377	0.580	0.245	0.374	0.586	0.244	0.373	0.588
		5	0.113	0.160	0.910	0.098	0.141	0.930	0.090	0.127	0.943	0.083	0.126	0.944	0.060	0.104	0.962
	Tansig	1	0.186	0.257	0.796	0.187	0.257	0.797	0.187	0.254	0.803	0.168	0.241	0.821	0.167	0.234	0.832
		2	0.148	0.228	0.831	0.133	0.207	0.860	0.130	0.188	0.884	0.119	0.173	0.902	0.125	0.178	0.897
		3	0.117	0.179	0.914	0.099	0.165	0.927	0.094	0.146	0.943	0.086	0.139	0.948	0.088	0.137	0.949
		4	0.272	0.412	0.497	0.250	0.378	0.576	0.246	0.376	0.582	0.247	0.375	0.584	0.246	0.373	0.587
		5	0.110	0.159	0.912	0.088	0.136	0.935	0.083	0.128	0.943	0.077	0.121	0.948	0.071	0.109	0.958
	Radbas	1	0.183	0.257	0.798	0.177	0.250	0.809	0.179	0.248	0.811	0.156	0.227	0.842	0.159	0.229	0.839
		2	0.150	0.239	0.814	0.134	0.202	0.867	0.125	0.182	0.892	0.127	0.180	0.895	0.127	0.182	0.892
		3	0.127	0.182	0.892	0.105	0.153	0.937	0.099	0.154	0.936	0.089	0.141	0.947	0.076	0.133	0.953
		4	0.282	0.411	0.501	0.264	0.381	0.569	0.271	0.412	0.498	0.254	0.378	0.578	0.240	0.371	0.591
		5	0.118	0.181	0.885	0.090	0.135	0.936	0.088	0.136	0.935	0.064	0.109	0.958	0.081	0.121	0.949
Scaled conjugate gradient	Logsig	1	0.187	0.258	0.796	0.178	0.251	0.806	0.185	0.261	0.791	0.189	0.272	0.772	0.219	0.317	0.691
		2	0.148	0.227	0.831	0.148	0.220	0.843	0.150	0.231	0.826	0.179	0.254	0.790	0.148	0.216	0.848
		3	0.121	0.192	0.901	0.114	0.177	0.916	0.108	0.161	0.930	0.104	0.176	0.916	0.087	0.153	0.937
		4	0.287	0.415	0.491	0.298	0.417	0.484	0.297	0.406	0.512	0.289	0.419	0.479	0.288	0.414	0.491
		5	0.112	0.162	0.908	0.107	0.158	0.912	0.095	0.143	0.928	0.112	0.159	0.911	0.157	0.217	0.835
	Tansig	1	0.190	0.260	0.793	0.185	0.257	0.797	0.186	0.255	0.800	0.174	0.243	0.818	0.177	0.257	0.797
		2	0.154	0.230	0.827	0.152	0.225	0.834	0.153	0.220	0.842	0.145	0.216	0.847	0.145	0.225	0.835
		3	0.114	0.179	0.914	0.097	0.165	0.926	0.110	0.173	0.920	0.114	0.177	0.915	0.085	0.153	0.937
		4	0.271	0.415	0.489	0.251	0.376	0.580	0.254	0.383	0.566	0.248	0.375	0.584	0.246	0.379	0.574
		5	0.114	0.162	0.908	0.112	0.159	0.911	0.104	0.153	0.918	0.094	0.142	0.930	0.105	0.151	0.920
	Radbas	1	0.185	0.262	0.790	0.188	0.260	0.792	0.189	0.273	0.771	0.192	0.262	0.789	0.183	0.250	0.808
		2	0.154	0.242	0.809	0.138	0.219	0.843	0.156	0.232	0.824	0.135	0.195	0.876	0.144	0.205	0.862
		3	0.125	0.201	0.891	0.103	0.154	0.937	0.088	0.147	0.942	0.105	0.165	0.927	0.164	0.241	0.843
		4	0.291	0.420	0.477	0.283	0.417	0.486	0.296	0.442	0.420	0.290	0.419	0.480	0.262	0.393	0.542
		5	0.125	0.206	0.851	0.110	0.161	0.909	0.101	0.146	0.925	0.079	0.124	0.946	0.084	0.133	0.938

**Table 7:** MLR performance from different types of model specification during training and testing.

Dataset	Type	Training			Testing		
		MAE	RMSE	R^2^	MAE	RMSE	R^2^
1	Linear	0.269	0.372	0.570	0.272	0.367	0.590
2		0.269	0.372	0.570	0.272	0.367	0.590
3		0.236	0.321	0.660	0.245	0.334	0.700
4		0.323	0.435	0.410	0.322	0.435	0.440
5		0.249	0.338	0.670	0.228	0.298	0.690
1	Interaction	0.219	0.327	0.670	0.227	0.325	0.680
2		0.219	0.327	0.670	0.227	0.325	0.680
3		0.152	0.239	0.820	0.162	0.237	0.850
4		0.317	0.431	0.420	0.311	0.423	0.470
5		0.157	0.244	0.830	0.140	0.190	0.870
1	Pure quadratic	0.183	0.264	0.780	0.187	0.265	0.780
2		0.183	0.264	0.780	0.187	0.265	0.780
3		0.117	0.185	0.890	0.119	0.188	0.900
4		0.295	0.420	0.450	0.283	0.407	0.510
5		0.119	0.193	0.890	0.109	0.163	0.910
1	Quadratic	0.181	0.254	0.800	0.183	0.254	0.800
2		0.181	0.254	0.800	0.183	0.254	0.800
3		0.106	0.172	0.900	0.111	0.177	0.920
4		0.300	0.406	0.480	0.292	0.397	0.530
5		0.107	0.172	0.910	0.103	0.155	0.920

## Data Availability

Not applicable.
